# Rubber band-assisted closure of a mucosal defect following duodenal EMR

**DOI:** 10.1016/j.vgie.2021.06.003

**Published:** 2021-07-10

**Authors:** Sarah S. Al Ghamdi, Zryan Shwani, Saowanee Ngamruengphong

**Affiliations:** Division of Gastroenterology and Hepatology, Johns Hopkins Hospital, Baltimore, Maryland

**Keywords:** AE, adverse event

## Abstract

Video 1 Rubber band–assisted closure of a mucosal defect after duodenal EMR.

Rubber band–assisted closure of a mucosal defect after duodenal EMR.

## Background

EMR of large duodenal adenomas is a technically challenging procedure. It carries a particularly high risk of perforation (5 % -10%) because of its relatively thin and fixed wall and delayed bleeding (15%) due to its rich vascular supply.^1^ Systematic closure of mucosal defects after duodenal endoscopic resection has been found to significantly reduce the risk of delayed adverse events (AEs) by 80%, specifically the risk of delayed bleeding and perforation.^2^ However, owing to the fixed retroperitoneal descending duodenum, apposition of the 2 mucosal edges is not always feasible, making closure using standard clips challenging. We describe a simple and safe technique using an inexpensive and widely available rubber band and standard endoscopic clips (Video 1, available online at www.giejournal.org).

## Technique

**Figure 1:**
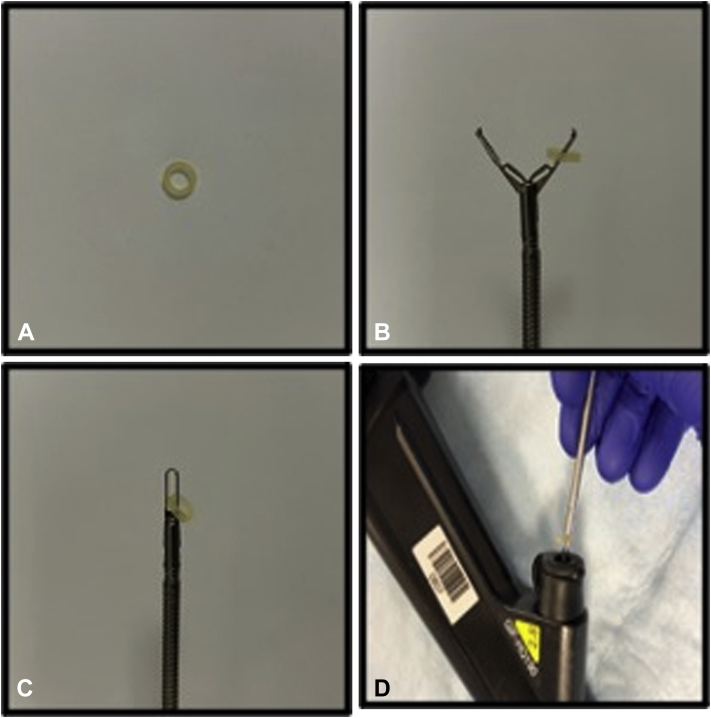
**A,** Rubber band used for closure. **B,** Rubber band is placed on 1 clip arm. **C,** Clip is closed securely. **D,** Clip with the attached rubber band is inserted into the endoscope instrument channel.

This technique requires the use of a commercially available orthodontic rubber band (Fig. 1A) in addition to standard endoscopic clips. When ready for use, a rubber band is placed on 1 clip arm (Fig. 1B) and the clip is securely closed (Fig. 1C). The clip, with its attached rubber band, is then inserted into the endoscope instrument channel (Fig. 1D). The clip is then positioned and deployed on the edge of the lesion at the area of maximal width, fixing the rubber band in place. Care should be taken to slowly open the clip to avoid dislodgment of the rubber band. A second clip is then used to grasp the rubber band and is secured onto the opposite edge of the mucosal defect, approximating both edges. This allows for further closure of the defect using clips in a standard fashion.

## Case presentation

We demonstrate this technique in a 72-year-old woman who was referred for evaluation of iron deficiency anemia. EGD was performed for evaluation and revealed a 3-cm × 3-cm flat polyp (Paris classification 0-IIa) in the second portion of the duodenum, sparing the ampulla. Capsule endoscopy and colonoscopy were subsequently performed and revealed no other polyps or etiology for anemia. The decision was made to proceed with EMR. The polyp was lifted using a mixture of 6% hetastarch and methylene blue. Complete piecemeal EMR was performed using snare cautery.

After resection, post-EMR defect margins were prophylactically treated with snare tip soft coagulation to help prevent recurrence.^3^^,^^4^ To reduce the risk of delayed AEs, closure of the EMR defect with endoscopic clips was attempted. Because of the large size of the resection bed, complete apposition of the 2 edges of the mucosal defect using standard endoscopic clips was not feasible. The decision was made to proceed with rubber band–assisted closure. As described, a small rubber band was attached to the first clip. The clip was then placed on the proximal edge of the lesion at the area of maximal width, fixing the rubber band in place. A second clip was used to grasp the rubber band and was then attached to the opposite edge of the defect, approximating both edges. This allowed for subsequent closure of the remaining mucosal defect by easily adding additional clips at the now approximated edges.

A total of 8 endoclips were used, achieving excellent closure. The patient was discharged home the same day. There were no AEs, including bleeding or delayed perforation. Final pathology revealed a tubulovillous adenoma with no high-grade dysplasia. Surveillance EGD 6 months later revealed no evidence of recurrent adenomatous tissue.

## Conclusions

The rubber band technique is a safe and easy technique using an inexpensive and widely commercially available device to help achieve closure of large mucosal defects, particularly those in challenging locations such as the duodenum.^5^

## Disclosure

*Dr Ngamruengphong is a consultant for Boston Scientific. All other authors disclosed no financial relationships.*

## Supplementary data

Video 1 Rubber band–assisted closure of a mucosal defect after duodenal EMR.
